# A System of Legal Medicine

**Published:** 1894-07

**Authors:** 


### BOOK NOTICES.

A SYSTEM OF LEGAL MEDICINE. A complete work of reference for medical and legal practitioners. By Allan McLane Hamilton, M. D., Consulting Physician to the Insane Asylum, of New York City, etc., etc. Assisted by Lawrence Godkin, Esq., of the New York Bar, and a corps of thirty collaborators, in its various departments, with which their scientific reputation is identified. E. B. Treat, Publisher, 5 Cooper Union, New York. In substantial cloth binding, per volume, $5.50 ; in full sheep, uniform law style, per volume, $6.50. Sold by subscription. Orders taken only for the complete work.

The list of contributors to this great work includes the names of some of the most distinguished writers and authorities upon Medical Jurisprudence in America. As a book of reference it will be found an invaluable help to medical men and by those of the legal profession who desire the aid of the most advanced and sound opinions of practical students of forensic medicine. So much opprobrium has been attached to the word "expert" that the spirit which so often impels men to go into court and become ardent partisans, finds no place in this system, and it will be the aim of the editor and his colleagues to give the work a decided judicial and impartial tone, so that it may be consulted with confidence by all as an authority of the first order.

Until recently the contributions in the United States to the literature of Medical Jurisprudence have been exceedingly meagre, if we may except Beck's classical but antiquated treatise, and other works limited in scope. For some time it has been the fashion to consult foreign books which are written for the benefit of trans-Atlantic readers, and in many respects are inapplicable to our methods, and not in conformity with the legal usages of this country. Consequently the appearance of an American treatise of this character will be especially timely and welcome.

A feature of the book will be the introduction of short articles upon special subjects prepared by distinguished members of the American bar which will form appendices to the different articles.

The legal gentlemen, who have been invited to write articles upon subjects with which they are especially familiar, have in most instances acted in conjunction with a medical collaborator.

The editor has aimed to make the work under consideration a repository of the most advanced ideas and valuable cases, and, except when the latter are unique, indispensable or especially pertinent, it will be his aim and that of his associates to avoid thread-bare material, and to illustrate the articles by new examples. The scope of the work is necessarily very great, but it is trusted that its contents will be found to be practical and concise. Extraneous matter is dispensed with, and the reader will be spared dry and uninteresting details and valueless decisions. A feature of "Hamilton's System of Legal Medicine" will be the presentation of a large amount of new experimental research.

The work will be comprised in two large royal octavo volumes, of about seven hundred pages each; illustrated when practicable and desirable by photographic reproductions from nature and other drawings and special diagrams; by chromo-lithography and engravings in line and half-tone process.

The first volume is now issued and lies before the editor. It is a superb work and abundantly fulfills the promise of the publisher that the mechanical execution—*paper, press-work and binding*—will be equal to the best known to the art of book-making.

